# Plant traits and environment: floating leaf blade production and turnover of *Nymphoides peltata* (S.G. Gmel.) O. Kuntze (Menyanthaceae)

**DOI:** 10.7717/peerj.13976

**Published:** 2022-09-01

**Authors:** Peter F. Klok, Gerard van der Velde

**Affiliations:** 1 Department of Animal Ecology and Physiology, Research Institute for Biological and Environmental Sciences (RIBES), Radboud University, Nijmegen, Netherlands; 2 Department of Particle Physics, Institute for Mathematics, Astrophysics and Particle Physics, Radboud University, Nijmegen, Netherlands; 3 Naturalis Biodiversity Center, Leiden, Netherlands

**Keywords:** Nymphaeid growth form, Leaf area, Leaf biomass, Leaf life span, Enclosure, Mesocosm, Damage, Floods, Water temperature, Nutrient limitation

## Abstract

**Background:**

Nymphaeid macrophytes, rooting in the sediment of water bodies and characterized by floating leaves, play an important role in wetland ecosystems. The present research deals with the effects of limited space, limited nutrient availability, water temperature and an unexpected inundation on the production, turnover and plasticity of floating leaves of the globally widespread species *Nymphoides peltata* (Fringed waterlily).

**Methods:**

The effects of these environmental conditions were studied in two plots in outdoor concrete tanks (CT1, CT2, mesocosms simulating occurrence in small ponds) and in two plots in the floodplain oxbow lake Bemmelse Strang (BS1, BS2). Plot CT1 was situated in a stand coexisting with helophytes, plot CT2 in a monospecific stand, plot BS1 in the center and plot BS2 at the open water border of a monospecific stand. All floating leaf blades within the plots were marked at appearance at the water surface and subsequently length, width and damage of each leaf and maximum and minimum water temperatures were measured bi-weekly. Area and biomass of leaf blades were calculated based on leaf length and width and were used to calculate turnover rates and production.

**Results:**

The growth period started in May and ended mid-October with continuous production of floating leaves during nearly the whole vegetation period. In the tanks the water level was very stable, but the lake underwent an inundation by river water, causing a sudden loss of existing leaves. Considering environmental conditions and based on the assumed ranking from low to high nutrient availability, the ranking of the plots was CT1, CT2, BS1, BS2. This order was found for maximum leaf life span and maximum leaf length, and the reverse order was found for number of leaves, new leaves per day and duration of the vegetation period. Turnover rates appeared to be relatively similar for plots CT1, CT2 and BS1, but for the deeper border plot BS2 lower ratios were found. These results indicate that increased enclosure with expected nutrient limitation causes (1) the production of high numbers of small leaves with larger totals for leaf area and biomass, (2) a shift towards increased sexual reproduction by the production of more flowering stem leaves.

## Introduction

### Distribution

*Nymphoides peltata* (S.G. Gmel.) O. Kuntze is a globally widespread aquatic plant species occurring as native in central, western and southern Europe, northern and western Asia, Kashmir, the Himalayas and Japan (Glück, 1924; Stuckey, 1974; Meusel, Jäger & Weinhert, 1978), and as invasive in North America (Stuckey, 1974; Darbyshire & Francis, 2008) and South Africa (Cheek, 2018). In the U.S. *N. peltata* was exterminated in drinking water reservoirs (Lamb et al., 2021). In New Zealand it was considered an invasive pest and it was exterminated (CABI Invasive Species Compendium, 2005). In Japan (Takagawa, Nishihiro & Washitai, 2005) and Poland (Urbisz & Urbisz, 1998) it is a protected species. The northern limit of occurrence of *N. peltata* coincides more or less with the 16 °C July isotherm (Van der Velde, Giesen & Van der Heijden, 1979).

### Growth form and importance of floating leaves

**Figure 1:**
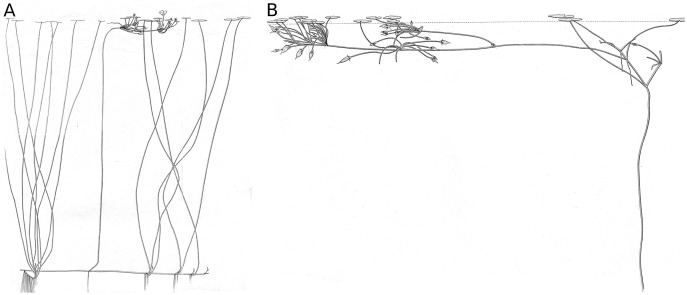
The nymphaeid growth form of *Nymphoides peltata*. The nymphaeid growth form of *Nymphoides peltata*. (A) Plant with short and long shoots bearing short shoot leaves and a flowering stem, (B) flowering stem with fruits and flowering stem leaves floating on the water surface. Drawing by T. C. Oor.

*Nymphoides peltata* is a macrophyte with a nymphaeid growth form (Luther, 1983; Den Hartog & Van der Velde, 1988) (Fig. 1). The plant possesses short shoots (rhizomes) rooted in the sediment from where petioles develop which hold the nearly orbicular leaf blades (laminae) at the water surface, and long shoots creeping over the sediment surface which act as runners to create distant short shoots (Van der Velde, Giesen & Van der Heijden, 1979). In this way it can expand rapidly in a vegetative way. Flowering stems develop from leaf axils of a long shoot and grow towards the water surface, developing short internodes with floating leaves by sympodial branching, forming a platform that holds the flower buds near the water surface. From this platform the flowers rise above the water surface to be pollinated by insects for the production of seeds (Van der Velde & Van der Heijden, 1981). Floating leaves with their petioles contribute most to the biomass production of *N. peltata* (Brock et al., 1983a). Floating leaf blades are important for the plant by processes such as photosynthesis (Smits et al., 1988; Yu et al., 2014), gas ventilation (Grosse & Mevischutz, 1987; Grosse & Bauch, 1991), chemical accumulation (*e.g*., heavy metals by hydropotes (Brock, 1983; Lavid et al., 2001)) and suppression of other aquatic macrophytes by shadowing (Larson, 2007; Zhu et al., 2019). Furthermore, floating leaf blades function as substratum for particular organisms (periphyton, sessile animal eggs), free moving species at and near the water surface, as isles in the open water for air-breathing animals, as substratum and nutrient for fungi and microbes and as food for more or less specialized invertebrates and for vertebrates. By their growth form, rooted plants with floating leaves structure the nymphaeid-dominated system in a particular way (Van der Velde, 1980), different from those by other growth forms of macrophytes (Den Hartog & Van der Velde, 1988).

### Habitat and environmental conditions

According to Bornette & Puijalon (2011) environmental characteristics of aquatic habitats rule macrophyte species occurrence, life-history traits and community dynamics among aquatic macrophytes. Important factors mentioned are light, temperature, water nutrient content, substrate characteristics, water movements and disturbances like floods and drawdowns.

In the Netherlands *N. peltata* is commonly found in ditches, ponds, lakes, canals, lowland brooks and small rivers, but not in moorland pools as the plant needs calcium for leaf development (Van der Velde, Custers & De Lyon, 1986; Smits, Schmitz & Van der Velde, 1992). The plant grows in particular in eutrophic, alkaline fresh water bodies (Grote, 1980; Casper & Krausch, 1981; Smits et al., 1988) on mineral soils, in particular clay (Döhler, 1963; Westhoff et al., 1971), till a depth of about 3 m, with an optimum growth at a depth of 20–150 cm (Funke, 1951). By severe wave action the plant is often restricted to the edges of large water bodies (Brock, 1985). Although *N. peltata* is also found growing on mineral soils with organic matter (Grote, 1980; Van der Velde, Custers & De Lyon, 1986), its tolerance for organic matter is lower than that of waterlilies (Van der Voo & Westhoff, 1961; Westhoff et al., 1971; Smits et al., 1990). The species occurs commonly in backwaters of large rivers which are regularly flooded by river water in winter (Van der Voo & Westhoff, 1961). Extremely low water levels and emergence are tolerated by *N. peltata* and lead to massive germination of seeds present in the sediment. The seedlings can quickly grow towards full grown plants (Brock, Van der Velde & Van de Steeg, 1987). The plant also adapts easily to a terrestrial way of life (Li et al., 2010a; Li, Yu & Xu, 2010b; Yu et al., 2014) and can even be cultured as a pot plant (Heimans & Thijsse, 1956). Emergent leaves show a longer life span than floating leaves (Tsuchiya, 1988). Stress by main flow and turbulence is better tolerated by *N. peltata* than by completely submerged macrophytes (Asaeda & Rashid, 2017). The same is true for mechanical damage as a result of boating and other recreational activities, fishing and aquatic management regimes, and perturbations by propellers and rakes in the upper water layer. *Nymphoides peltata* shows a high capacity for foliage compensation at such disturbances (Cao, Liu & Wang, 2016) and it shows a high plasticity (Xiao et al., 2011).

### Research question and hypothesis

The research question for this basic study is:

How are floating leaf blade characteristics of *N. peltata* influenced by different environmental conditions?

The hypothesis is posed that *N. peltata* recycles nutrients more frequently in dense stands than in pioneer or border stands, because a high consumption of nutrients during the growing season will lead to shortages for development and growth with consequences for leaf development. Similarities in development indicate stable traits, while differences must be interpreted as adaptations or responses to the environment. The study will furthermore be used as a basis for studying connected processes such as decomposition.

Floating leaves are indicated as leaves further in this article.

## Materials and Methods

### Sites

Research took place in four plots with a size of 50 × 50 cm (0.25 m^2^) each. Pilot observations proved this size sufficient to follow the leaf development in a *Nymphoides* stand in a representative way. Plots CT1 and CT2 were situated in outdoor concrete tanks (CT) on the site of Radboud University Nijmegen (coordinates 51°49′21″N, 5°52′23″E) and plots BS1 and BS2 in Bemmelse Strang (BS), a fresh water oxbow lake north of the River Waal near Nijmegen (coordinates 51°52′43″N, 5°53′03″E).

The concrete tanks were sunken in the ground, surrounded by a paved area and covered by a frame with chicken wire to prevent leaves and animals falling into the water adding nutrients. The inner surface area of the tanks measured 80 × 150 cm with a depth of 60 cm. The tanks were filled with river water. A heavy river clay mud layer on the bottom resulted in a water depth of 40–50 cm and an alkaline and eutrophic water quality. The water level fluctuated slightly due to precipitation and evaporation and there was no wave and wind action. Plot CT1 contained a mixed culture of *N. peltata*, *Glyceria fluitans* (L.) R. Br. and *G. maxima* (Hartm.) Holmb. and plot CT2 a monoculture of *N. peltata*. The *Glyceria* species spontaneously occurred and all plants inclusive *N. peltata* in the tanks developed for 3 years prior to this research. No additional water or nutrients were added to the tanks.

Bemmelse Strang is a shallow, isolated, alkaline (2.6–4.8 meq.L^−1^), and eutrophic oxbow lake (pH 7.6–8.6), a former branch of the River Waal. The bottom of a large *Nymphoides* stand consisted of sand and a detritus layer with increasing thickness towards the littoral border of the lake. The hydrology was influenced by precipitation, evaporation, upward seepage and river water overflow. Plot BS1 was situated in the center of the stand and plot BS2 at the open water margin of the stand. The water depth fluctuated for BS1 from 30–106 cm (mean 67 cm, standard deviation 22 cm) and for BS2 from 82–154 cm (mean 117 cm, s.d. 22 cm). Minimum depth was measured on 20 October 1980, maximum depth on 7 August 1980. The *N. peltata* stands were exposed to moderate wave and wind action.

### Field data

**Figure 2:**
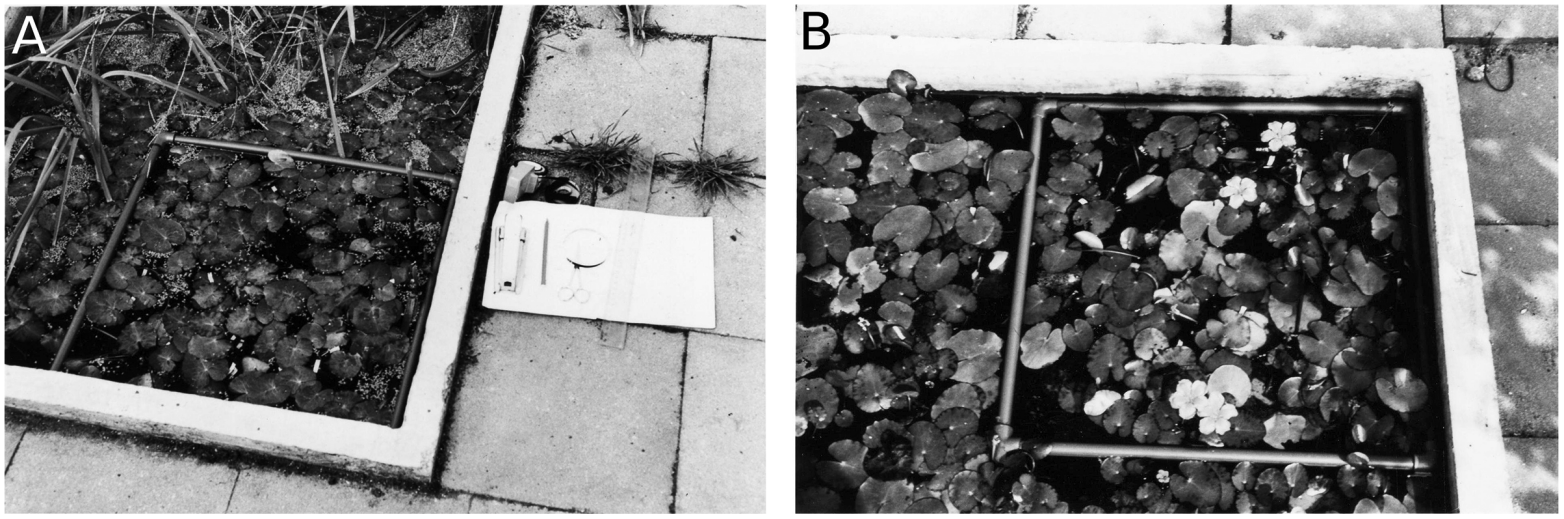
Concrete tanks with plots. Concrete tanks with removable floating 50 × 50 cm tube frame boundary for a *Nymphoides peltata* plot. (A) Plot with nearby helophytes (CT1), (B) monospecific plot (CT2).

A non-destructive leaf-marking method was used to mark all leaves originating within a plot at appearance, which enabled data collection during the complete life span of each leaf. During data collection in the tanks a square PVC tube frame was placed on the vegetation in a fixed corner of the tank (Fig. 2). In Bemmelse Strang a similar perforated tube frame was held permanently in a fixed position approximately 15 cm below the water surface by cork floaters and anchored by four bricks. By keeping the frame under water, the unrolling of leaves in the plot was not hindered. All leaves having their petioles within the frame were used for data collection.

Leaves were tagged by numbered Rotex tapes (width 12 mm) in the tanks and by small numbered aluminum strips in Bemmelse Strang. Tags were fixed around the petiole just below the leaf. Two leaf types were distinguished: short shoot leaves and flowering stem leaves.

A leaf was considered gone when the leaf had disappeared or when the remaining lamina totally sank under water. The presence over time of a floating leaf is called the leaf life span, calculated in days from the date of appearance at the water surface until the date it was gone. Terms used in other studies are leaf persistence (Brock et al., 1983a) and longevity (Chabot & Hicks, 1982).

Measurements and observations of all leaves within a plot took place twice a week during the growing season. It included tagging newly unrolled leaves, counting the actual number of leaves, measuring leaf length and width and visually estimating both leaf damage and decomposition as percentage of the potential leaf area of each leaf. Leaf length was measured from the leaf tip to one of the basal lobe tips.

A distinction was made between potential, actual and photosynthetic leaf area. The potential area is defined as the area of an entirely intact leaf, the actual area as the potential area minus the area missing (loss caused by consumption and other animal activities, by mechanical damage, by decay causing disintegration) and the photosynthetic area as the remaining green part of the actual area. The same distinction was made for leaf biomass.

Maximum and minimum water temperatures were collected bi-weekly in the plots by means of maximum-minimum thermometers at a depth of 10 cm as part of the standard data taking procedure. After each read-out the thermometers were reset and installed again in the water.

Data for CT1 and CT2 were collected in 1978 and 1979, data for BS1 and BS2 in 1980. Since some data of the first weeks in 1978 for CT1 and CT2 were missing and the growing season of *N. peltata* started on the same date in 1978 and 1979, leaf data and water temperature data of these weeks in 1979 have been used to complete the picture.

### Growth, vegetation and flowering stem leaf periods

The growth period of *N. peltata* of a plot was calculated as the number of days during which new leaves appeared at the water surface. The vegetation period was calculated as the number of days between the day of first leaf emergence and the day that all leaves had disappeared from the water surface. The flowering stem leaf period was calculated as the vegetation period for flowering stem leaves only.

### Regression equations for calculating leaf area and biomass

The leaf area was calculated from the leaf length and width by Eq. (1), determined by previous research (Van der Velde et al., 1982). The leaf biomass was calculated from the leaf area by regression Eq. (2) with coefficients determined from data of undamaged, randomly harvested green leaves outside the plots. Biomass is given in grams ash-free dry weight (g AFDW). Mathematically, the equations for potential area and for biomass are described by:

(1) $$A\left( {L,W} \right) = 1.028*\pi *{\left( {\displaystyle{{L + W} \over 4}} \right)^2}$$

(2) $$B_i(A)=d_{i,}A+e_i$$

where:

*A(L,W)* = potential leaf area at length *L* and width *W* (mm^2^)

*L* = leaf length (mm)

*W* = leaf width (mm)

1.028 = correction factor (the leaves are not circular)

*B*_*i*_*(A)* = potential leaf biomass in plot *i* for potential leaf area *A* (g AFDW)

A = potential leaf area (mm^2^)

*i* = plot

*d*_*i*_, *e*_*i*_ = coefficients for plot *i*

Since leaf samples for the calculation of biomass were collected monthly during this research, the regression Eq. (2) were calculated per month. Annual regression equations, including all monthly harvested leaves, were also calculated. Separate sets of regression equations were made for tank plots and for Bemmelse Strang plots (Table 1).

**Table 1:** Regression equations to calculate biomass from leaf area. Regression equations to calculate biomass from leaf area for *Nymphoides peltata* in tank and lake plots per leaf. Where *n* = number of leaves, A = area (cm^2^) and B = biomass (mg AFDW). Equations have been tested with the Student t-test. Coefficients are computed both for use in monthly periods and for annual use (Van der Velde et al., 1982).

Location	Period	*n*	Equation	SE	r^2^	*p*
Concrete tanks (CT1, CT2)	May–June	25	B = 2.956 * A – 2.120	13.66	0.96	<0.001
	July	25	B = 4.265 * A – 7.135	9.00	0.95	<0.001
	August	25	B = 3.267 * A + 1.033	11.79	0.85	<0.001
	September–October	25	B = 3.610 * A + 1.573	3.06	0.97	<0.001
	Annual	100	B = 3.018*A + 6.506	13.81	0.91	<0.001
Lake Bemmelse Strang (BS1, BS2)	May–June	20	B = 3.625 * A – 21.815	14.02	0.93	<0.001
	July	18	B = 4.439 * A – 37.529	15.81	0.96	<0.001
	August	19	B = 4.375 * A – 21.450	30.99	0.95	<0.001
	September	28	B = 3.901 * A – 27.868	15.40	0.95	<0.001
	October	21	B = 3.386 * A – 21.746	21.97	0.93	<0.001
	Annual	106	B = 3.925*A – 26.572	26.06	0.91	<0.001

For each sampling date the total potential area (leaf area index or LAI) and biomass of a plot were calculated by summation of the potential area and biomass values of the individual leaves in the plot on that date, respectively.

The total annual production of potential area was calculated by summation of the final potential areas after leaf growth of all produced leaves per plot. For conversion to potential biomass both monthly and annual regression equations were used. Actual and photosynthetic area and biomass were calculated from potential area and biomass by subtracting the field data loss percentages of leaf area per leaf per plot.

### Statistics

Since all data were stored in MS Excel (version 2009), this package was used to compute mean values, standard deviations, trend lines and perform single factor (or one-way) ANOVA tests.

## Results

Results are described by values of leaf blade characteristics, by the influence of water temperature, by the impact of an unexpected inundation on the characteristics, by development in time of characteristics and by ratios based on characteristics.

### Leaf blade characteristics

Leaf blade characteristics consist of data about number of leaves, leaf life span, vegetation period, growth period, flowering stem period, leaf length, leaf width and leaf area. Values of leaf blade characteristics of the four plots are listed in Table 2.

**Table 2:** Leaf characteristics of *Nymphoides peltata* per plot. Leaf characteristics of *Nymphoides peltata* per plot in concrete tanks (CT1, CT2) and Bemmelse Strang (BS1, BS2). Leaf biomass data are listed in Table 10.

Location Year		CT1 1978	CT2 1978	BS1 1980	BS2 1980
Number of leaves					
Total	m^−2^.yr^−1^	2,552	2,492	1,712	1,108
Short shoot	m^−2^.yr^−1^	2,148	1,644	1,664	1,096
Flowering stem	m^−2^.yr^−1^	404	848	48	12
Mean new per day	m^−2^	14.92	14.92	11.05	7.01
Maximum	m^−2^	608	832	392	380
Date of maximum		Aug. 15	Aug. 18	Jun. 16	Sep. 25
Leaf life span					
Maximum	d	43	54	59	63
Minimum	d	3	2	3	3
Mean	d	25.30	22.51	22.85	29.59
Standard deviation	d	7.64	9.06	10.93	13.26
Vegetation period					
Length	d	202	184	169	169
Begin date		May 2	May 6	May 18	May 18
End date		Nov. 20	Nov. 6	Nov. 3	Nov. 3
Growth period					
Length	d	171	167	155	158
Begin date		May 2	May 6	May 18	May 18
End date		Oct. 20	Oct. 20	Oct. 20	Oct. 23
Flowering stem leaf period					
Length	d	107	114	66	62
Begin date		Jun. 20	Jun. 13	Jul. 15	Aug. 26
End date		Oct. 5	Oct. 5	Sep. 19	Oct. 27
Leaf length (pot.)					
Maximum	cm	9.2	9.2	12.9	14.7
Minimum	cm	1.0	0.8	2.6	3.1
Range	cm	8.2	8.4	10.3	11.6
Mean begin	cm	4.09	4.17	6.11	6.84
Standard deviation	cm	1.46	1.84	1.59	1.60
Mean end	cm	4.41	4.42	7.22	8.14
Standard deviation	cm	1.57	2.00	1.87	2.08
Range	cm	0.32	0.25	1.12	1.30
Leaf width (pot.)					
Maximum	cm	8.0	9.0	10.9	11.3
Minimum	cm	0.8	0.7	2.4	2.9
Range	cm	7.2	8.3	8.5	8.4
Mean begin	cm	3.68	3.79	5.40	5.92
Standard deviation	cm	1.27	1.58	1.32	1.26
Mean end	cm	3.97	4.02	6.54	7.18
Standard deviation	cm	1.36	1.71	1.55	1.68
Range	cm	0.29	0.23	1.14	1.26
Leaf area					
Tot. pot.	m^2^.m^−2^.yr^−1^	3.5270	3.8204	6.9103	5.5477
Max. pot.	m^2^.m^−2^	1.4295	1.4274	1.7741	2.2719
Max act.	m^2^.m^−2^	1.4287	1.4168	1.5760	2.0489
Max. phot.	m^2^.m^−2^	0.9528	0.9326	1.4180	1.6752
Mean pot. per day	m^2^.m^−2^.d^−1^	0.0175	0.0208	0.0409	0.0328
Mean pot. per leaf	m^2^.m^−2^	0.0016	0.0017	0.0040	0.0050
Standard deviation	m^2^.m^−2^	0.0010	0.0013	0.0019	0.0023
Max. pot. date		Jul. 11	Jun. 30	Sep. 5	Oct. 20
Max act. date		Jul. 11	Jun. 30	Sep. 5	Sep. 16
Max. phot. date		Jun. 9	Jun. 30	Jun. 23	Sep. 16

### Water temperature, growth period and vegetation period

**Figure 3:**
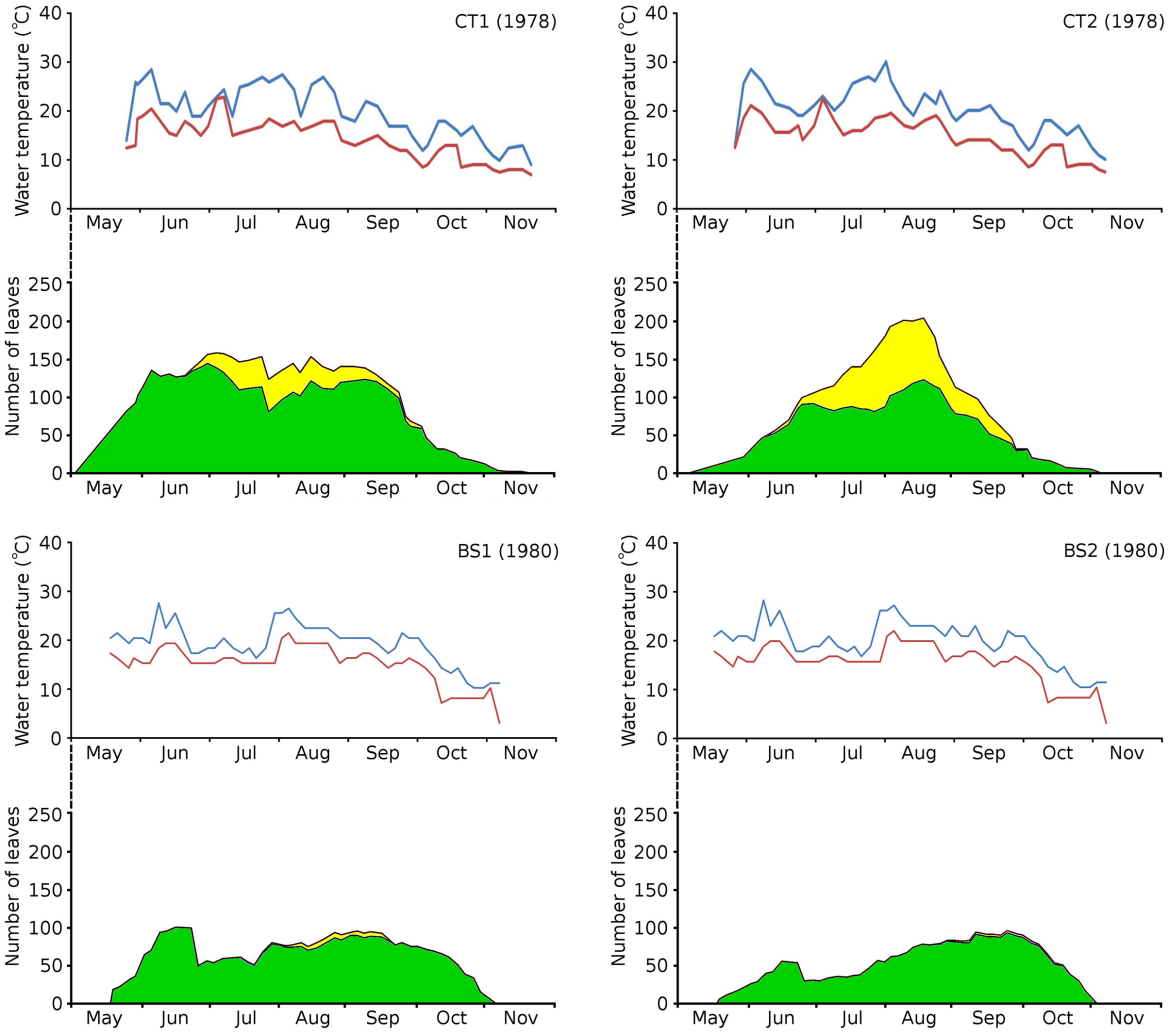
Water temperatures in the *Nymphoides peltata* plots. Water temperatures in the *Nymphoides peltata* plots, combined with the number of leaves per plot over time. Temperatures are blue for maximum (day) and red for minimum (night) values. The contribution to leaves per plot over time is divided in short shoot leaves (green) and flowering stem leaves (yellow).

**Figure 4:**
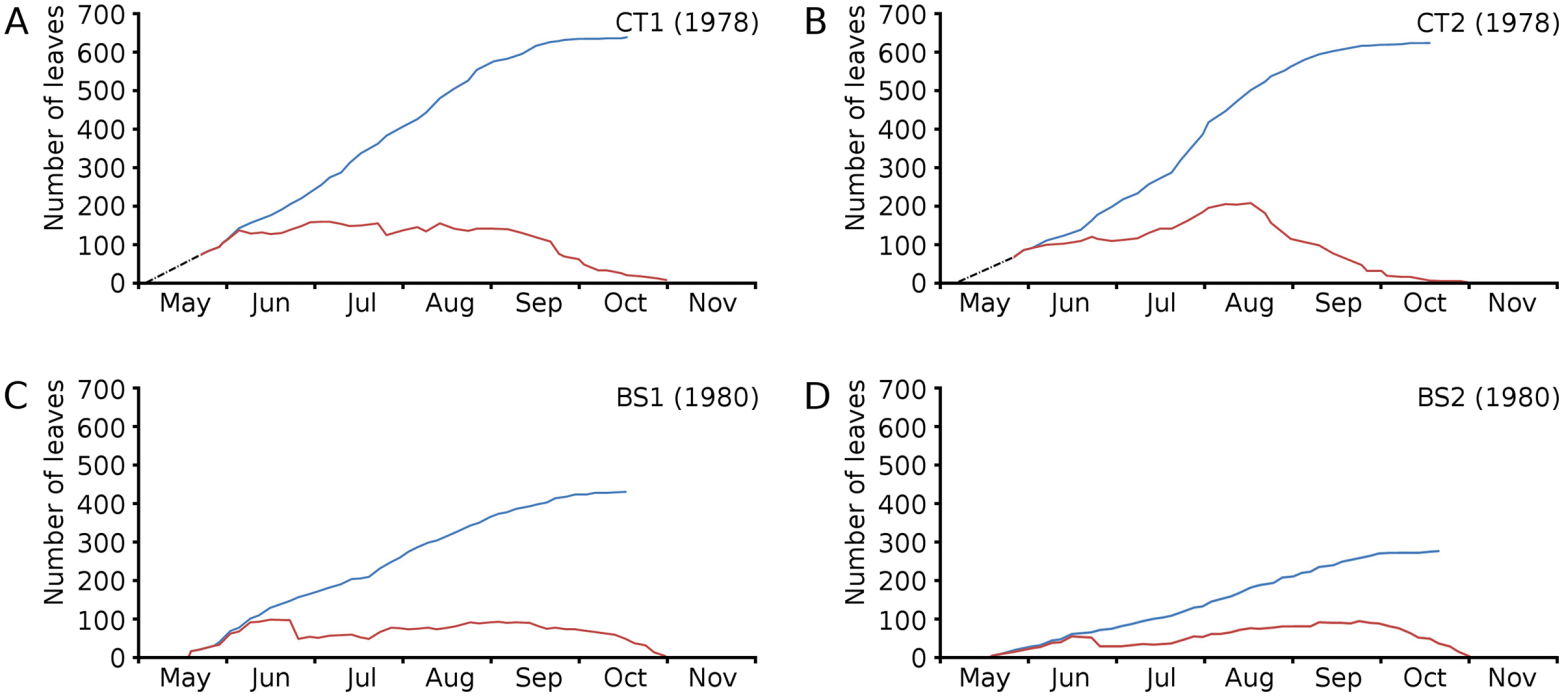
Numbers of floating leaves per plot over time. Number of floating leaves present (red line) and cumulative number of leaves (blue line) of *Nymphoides peltata* per plot over time. The dash-dotted black line indicates missing data in (A) and (B).

The growth period of *N. peltata* started in May at a daily water temperature max(min) of 12(7) °C in the tanks and of 20(17) °C in Bemmelse Strang and lasted until the second half of October when minimum water temperatures dropped below 10 °C (Fig. 3, Tables 3, and 4). In the tank plots the growth period started 2 weeks earlier than in the Bemmelse Strang plots, while the end date of the growth periods was similar for all plots. The production of new leaves during the growth period continued almost the whole vegetation period. Growth/vegetation period ratios for *N. peltata* were 84–94% (Fig. 4, Table 2).

**Table 3:** Water temperature data of measuring dates for growth and vegetation periods. Water temperature data of measuring dates for growth and vegetation periods of *Nymphoides peltata* in concrete tank and Bemmelse Strang plots. With CT1 (1978–1979), CT2 (1978–1979), BS1 (1980), BS2 (1980).

Plot	Daytime water temperature (°C)					Nighttime water temperature (°C)				
	max	min	range	average	s.d.	max	min	range	average	s.d.
	Growth period					Growth period				
CT1	28.50	12.00	16.50	20.84	4.67	23.00	6.50	16.50	14.73	3.72
CT2	30.00	12.00	18.00	20.26	4.51	22.50	6.50	16.00	14.72	3.67
BS1	27.00	11.00	16.00	19.52	3.37	21.00	7.00	14.00	15.48	3.11
BS2	27.00	10.00	17.00	19.40	3.66	21.00	7.00	14.00	15.31	3.27
	Vegetation period					Vegetation period				
CT1	28.50	9.00	19.50	19.69	5.33	23.00	6.50	16.50	13.85	4.15
CT2	30.00	10.00	20.00	19.63	4.87	22.50	6.50	16.00	14.19	3.94
BS1	27.00	10.00	17.00	18.77	4.10	21.00	3.00	18.00	14.79	3.83
BS2	27.00	10.00	17.00	18.85	4.14	21.00	3.00	18.00	14.79	3.83

**Table 4:** Water temperatures of the last three measuring dates before the end of the growth and the vegetation period. Water temperatures of the last three measuring dates before the end of the growth and the vegetation period of *Nymphoides peltata* as maximum(minimum) °C for plots CT1 (1978), CT2 (1978), BS1 (1980), BS2 (1980).

	Temperatures at end of growth period				Temperatures at end of vegetation period			
Plot	CT1	CT2	BS1	BS2	CT1	CT2	BS1	BS2
Last-but-two date	18(13)	18(13)	13(8)	14(8)	13(8)	13(9)	10(8)	10(8)
Last-but-one date	16(13)	16(13)	14(8)	11(8)	13(8)	11(8)	11(10)	11(10)
Last date	15(9)	15(9)	11(8)	10(8)	9(7)	10(8)	11(3)	11(3)

The vegetation period of all plots showed a max(min) daytime water temperature of 30(10) °C and a nighttime water temperature of 23(3) °C. The average water temperatures did not differ very much between the plots and ranged for daytime between 19.4–20.8 °C (growth period) and 18.9–19.7 °C (vegetation period) and for nighttime between 14.7–15.5 °C (growth period) and 13.9–14.8 °C (vegetation period) (Table 3).

### Inundation and leaf loss

**Figure 5:**
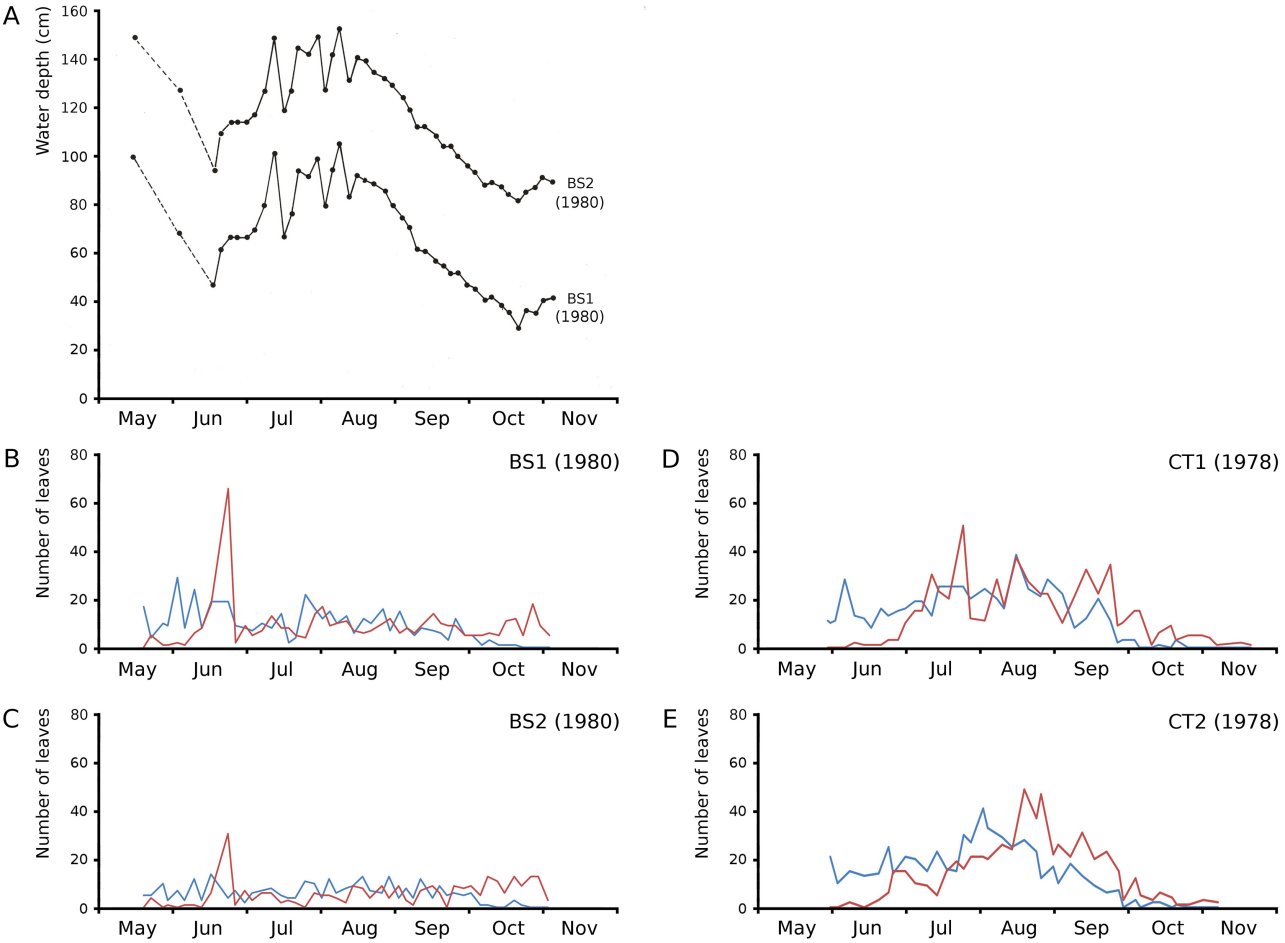
Impact of water level fluctuations. Water level fluctuations in Bemmelse Strang plots and leaf production and leaf loss over time for all *Nymphoides peltata* plots. (A) water level fluctuations, (B, C, D, E) leaf production (blue) and leaf loss (red).

On June 23, 1980, Bemmelse Strang was inundated unexpectedly by overflow of the River Waal by 0.5 m. The inundation period lasted until July 29, 1980, and led to water temperatures of on average 7 °C lower during the day and 4 °C during the night and to water level fluctuations with impact on leaf loss and production in plots BS1 and BS2. The loss of leaves of *N. peltata* by drowning was 49% of the leaves present in BS1 and 55% in BS2. The percentage of lost leaf area on June 23 compared to the total leaf area produced, was 17.01% for BS1 and 8.75% for BS2. The leaf loss peak of June 23 was followed by a month of decreased numbers of newly developed leaves. The concrete tanks CT1 and CT2, with only small fluctuations in water level, showed leaf loss peaks in July and at the end of August, respectively (Fig. 5).

### Development of leaves

Development of leaf blades is shown in figures by plots over time and by scatter plots of two variables with accompanying data in tables.

**Figure 6:**
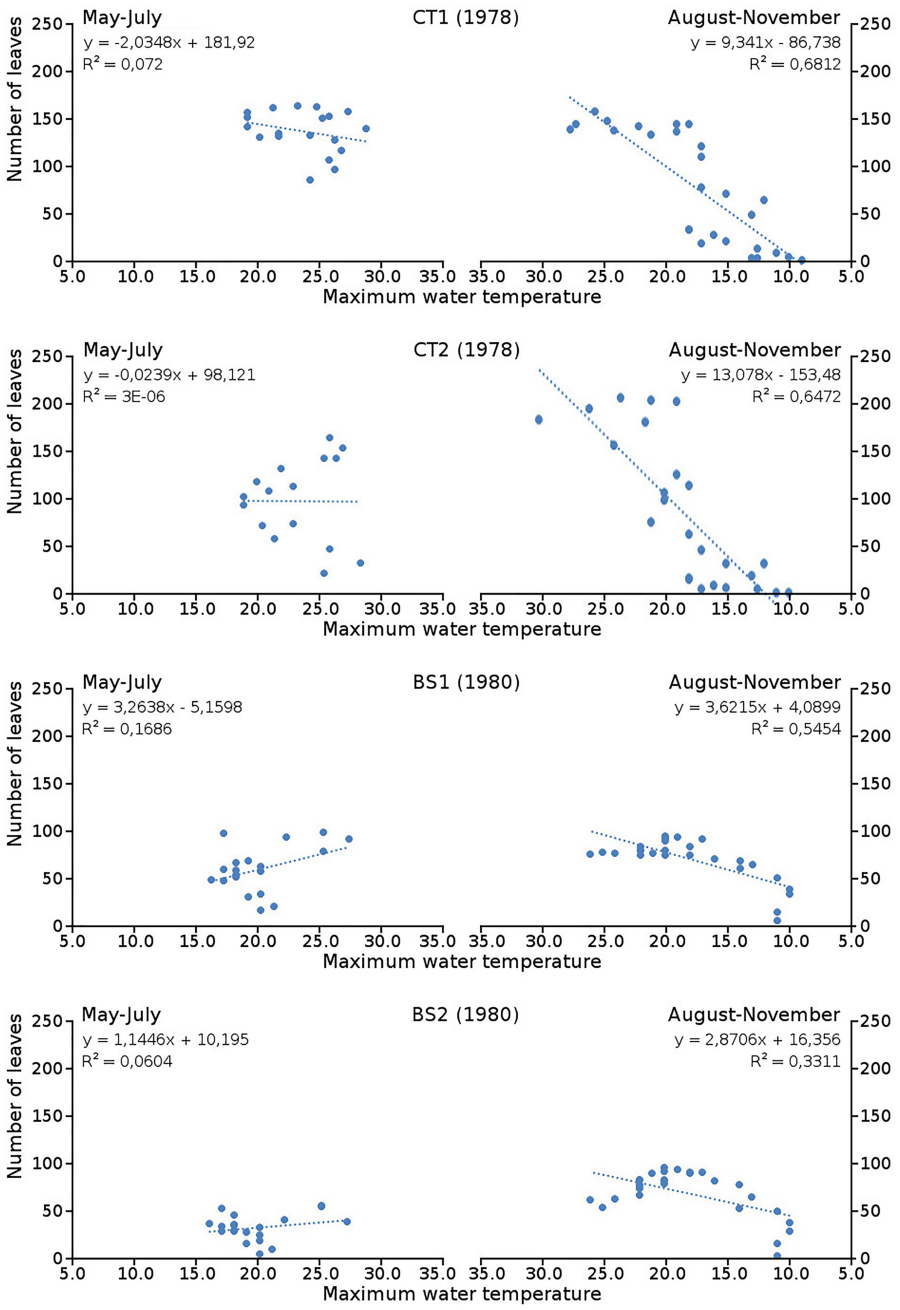
Relation between number of leaves and maximum water temperature. Relation between number of leaves and maximum water temperature on measuring dates with linear trend lines. Per plot each figure is divided in increasing temperatures from May through July (left half) and decreasing temperatures from August through November (right half).

**Figure 7:**
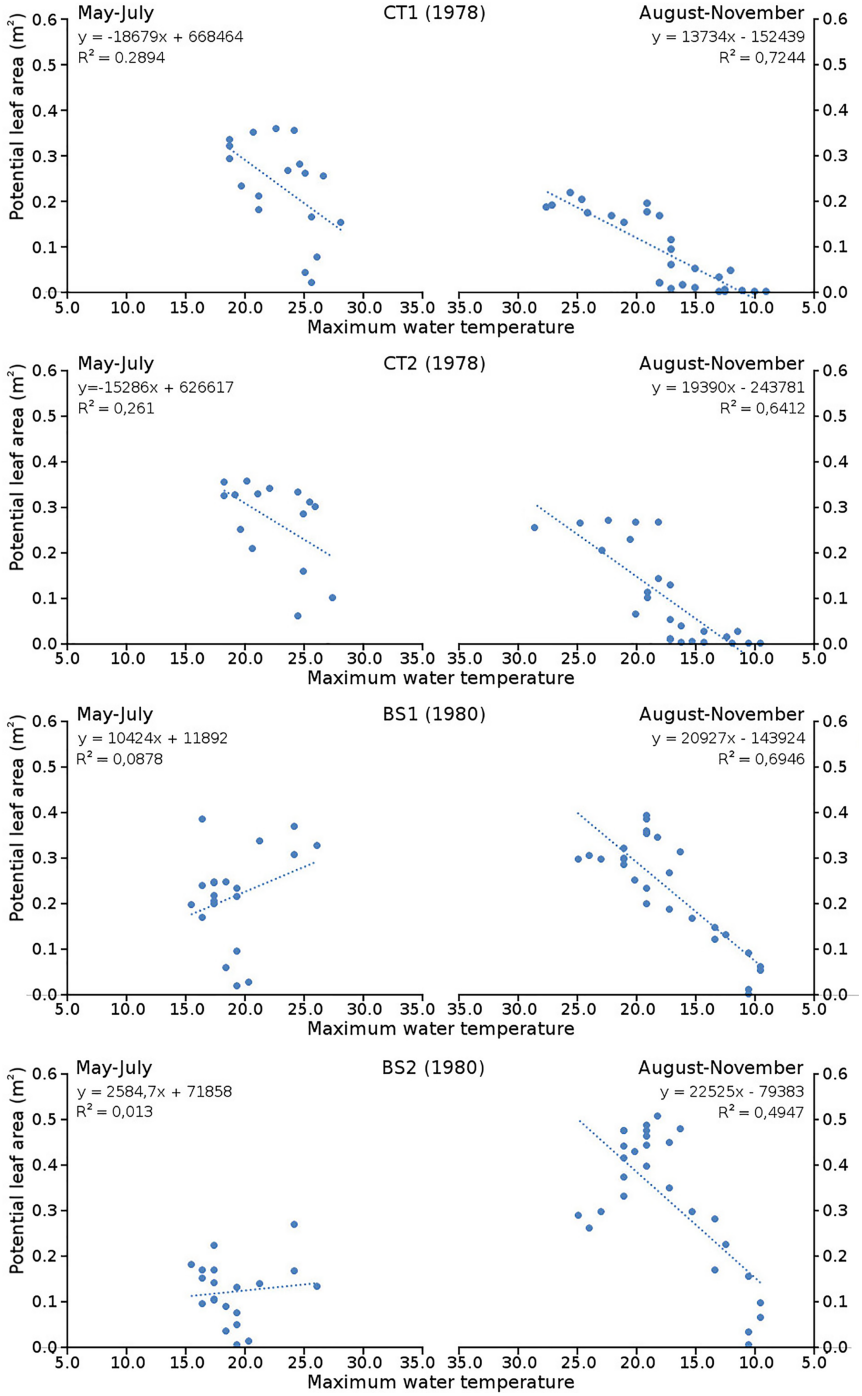
Relation between potential leaf area and maximum water temperature. Relation between potential leaf area and maximum water temperature on measuring dates with linear trend lines. Per plot each figure is divided in increasing temperatures from May through July (left half) and decreasing temperatures from August through November (right half).

Cumulative numbers of leaves per plot over time showed a regular increase over nearly the whole vegetation period. The actual numbers of leaves showed a rapid increase from the start of the growing season in May towards a relatively high number in June. Decrease of numbers started in September (BS1, BS2) or after a peak number in August (CT1, CT2). The actual number of all leaves, with separate contributions of short shoot and flowering stem leaves, is shown per plot over time in combination with the observed maximum and minimum water temperature over time. The number of leaves and the total potential leaf area in time showed a clear correlation with the course of the water temperature (Figs. 6 and 7). Development of leaves took place above 15 °C and leaves disappear when the water temperature dropped below 10 °C.

### Leaf life span and leaf size

Both leaf length frequencies and leaf life span frequencies of the four plots have been tested for differences by a single-factor ANOVA. Neither showed significant differences (leaf length *p*-value = 0.318 and leaf life span *p*-value = 0.548).

**Figure 8:**
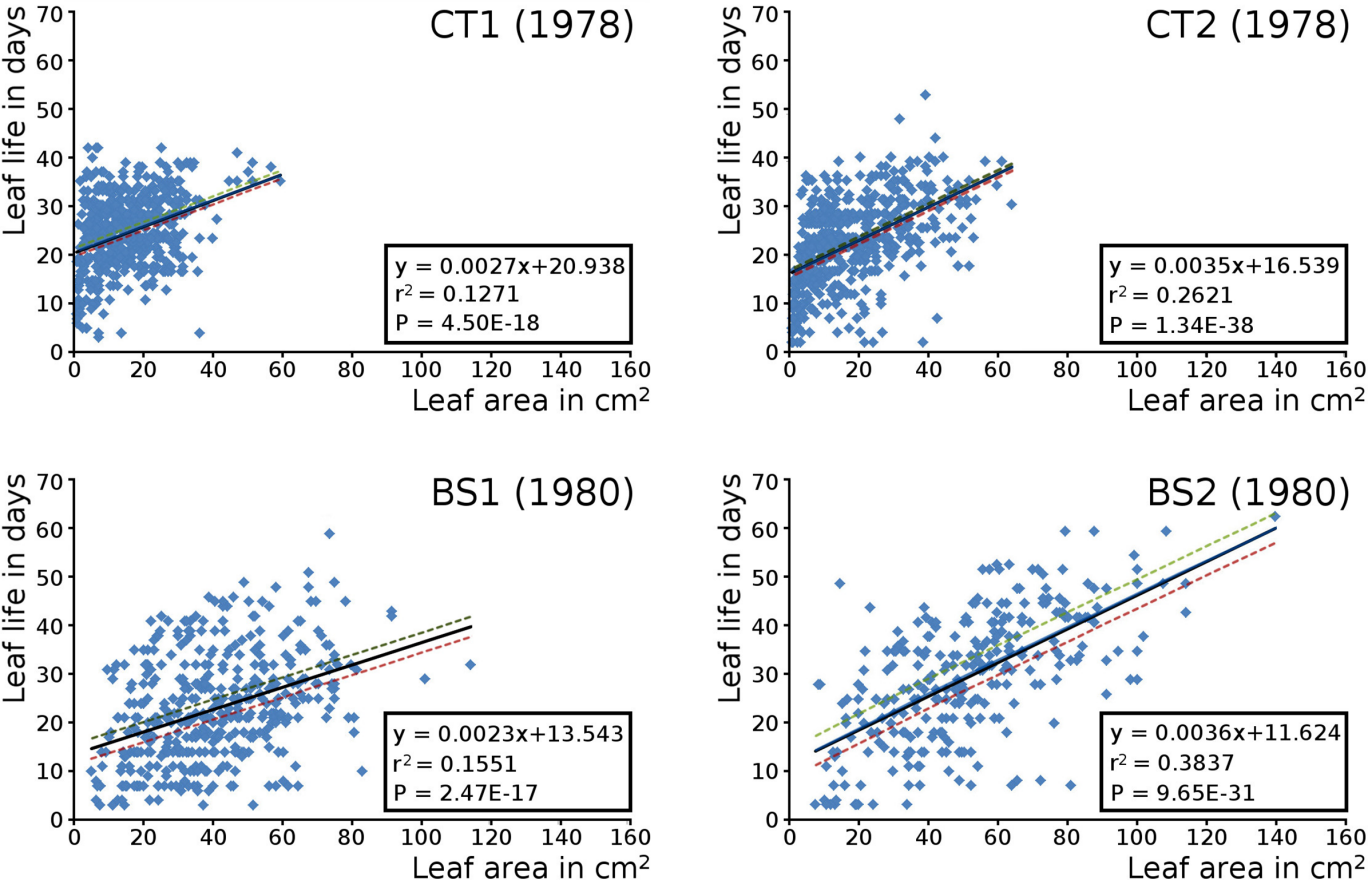
Maximum potential leaf area vs leaf life span. Scatter plots of maximum potential leaf area *vs*. leaf life span of all *Nymphoides peltata* leaves per plot with linear trend lines and 95% confidence interval. See Table 7 for confidence interval values.

The relation between the maximum potential leaf area and the leaf life span for all leaves per plot is shown by means of scatter plots with linear trend lines, showing a correlation of leaf size with leaf life span in all cases. Larger leaves existed generally longer than smaller leaves (Fig. 8).

Short shoot leaves were in each plot generally much larger than flowering stem leaves. The mean potential leaf area values of the plots ranking from low to high were CT1, CT2, BS1 and BS2 (Table 5). Confidence interval values have been computed for all leaves, and separately for short shoot and for flowering stem leaves (Table 6).

**Table 5:** Mean potential leaf area values of *Nymphoides peltata*. Mean potential leaf area values of *Nymphoides peltata* with standard deviations (s.d.) for all leaves, short shoot leaves and flowering stem leaves per plot.

Plot (year)		CT1 (1978)	CT2 (1978)	BS1 (1980)	BS2 (1980)
All	m^2^.m^−2^	0.0016	0.0017	0.0040	0.0050
s.d.		0.0010	0.0013	0.0019	0.0023
Short shoot	m^2^.m^−2^	0.0018	0.0023	0.0041	0.0050
s.d.		0.0009	0.0013	0.0019	0.0023
Flowering stem	m^2^.m^−2^	0.0006	0.0007	0.0020	0.0029
s.d.		0.0005	0.0006	0.0009	0.0018

**Table 6:** Confidence interval values of trend lines for maximum potential leaf area *vs*. leaf life per leaf. Confidence interval values of trend lines for maximum potential leaf area vs leaf life per leaf in *Nymphoides peltata* plots CT1 (1978), CT2 (1978), BS1 (1980) and BS2 (1980). Where all = all leaves, ssl = short shoot leaves, fsl = flowering stem leaves, followed by the number of leaves per square meter between brackets. See Fig. 9 for scatter plots and trend line equations for all leaves.

Plot	Leaves	Mean (mm^2^)	SE	95% conf int	Lower limit	Upper limit
**CT1**	all (555) ssl (454) fsl (101)	1,588.611 1,806.827 607.720	0.303 0.319 0.714	1.964 1.965 1.984	24.710 26.025 17.832	25.901 27.280 20.666
**CT2**	all (558) ssl (346) fsl (212)	1,711.665 2,344.753 678.417	0.330 0.407 0.505	1.964 1.967 1.971	21.859 24.440 17.053	23.154 26.041 19.044
**BS1**	all (428) ssl (416) fsl (12)	4,036.374 4,095.216 1,996.510	0.486 0.494 2.819	1.965 1.966 2.228	21.891 21.956 13.805	23.803 23.899 26.367
**BS2**	all (277) ssl (274) fsl (3)	5,006.969 5,030.387 2,868.123	0.627 0.614 1.600	1.969 1.969 12.706	28.358 28.131 26.069	30.826 30.549 66.736

### Leaf growth at the water surface

**Figure 9:**
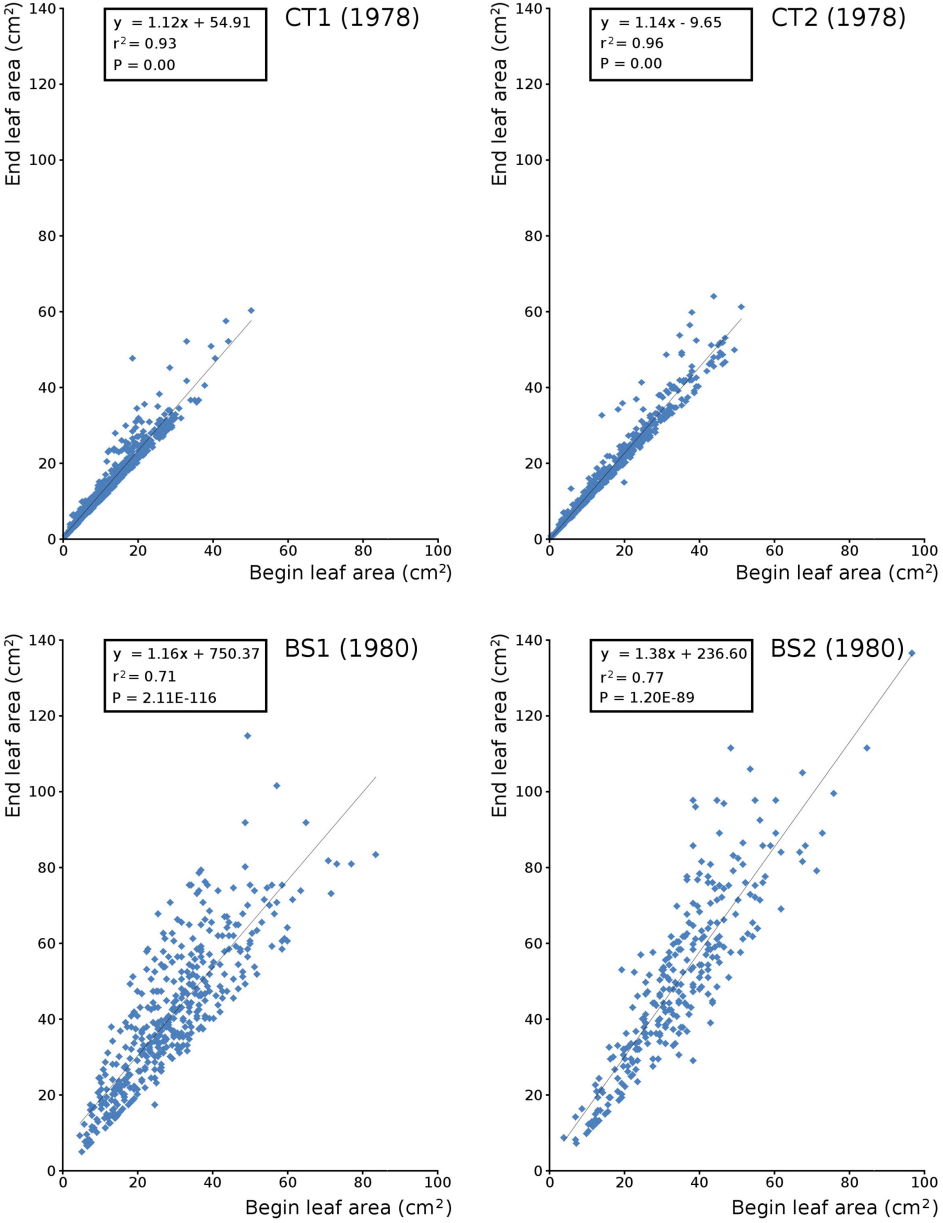
Relation between begin and end leaf area. Relation between begin and end leaf area of *Nymphoides peltata* with linear trend line per plot.

**Figure 10:**
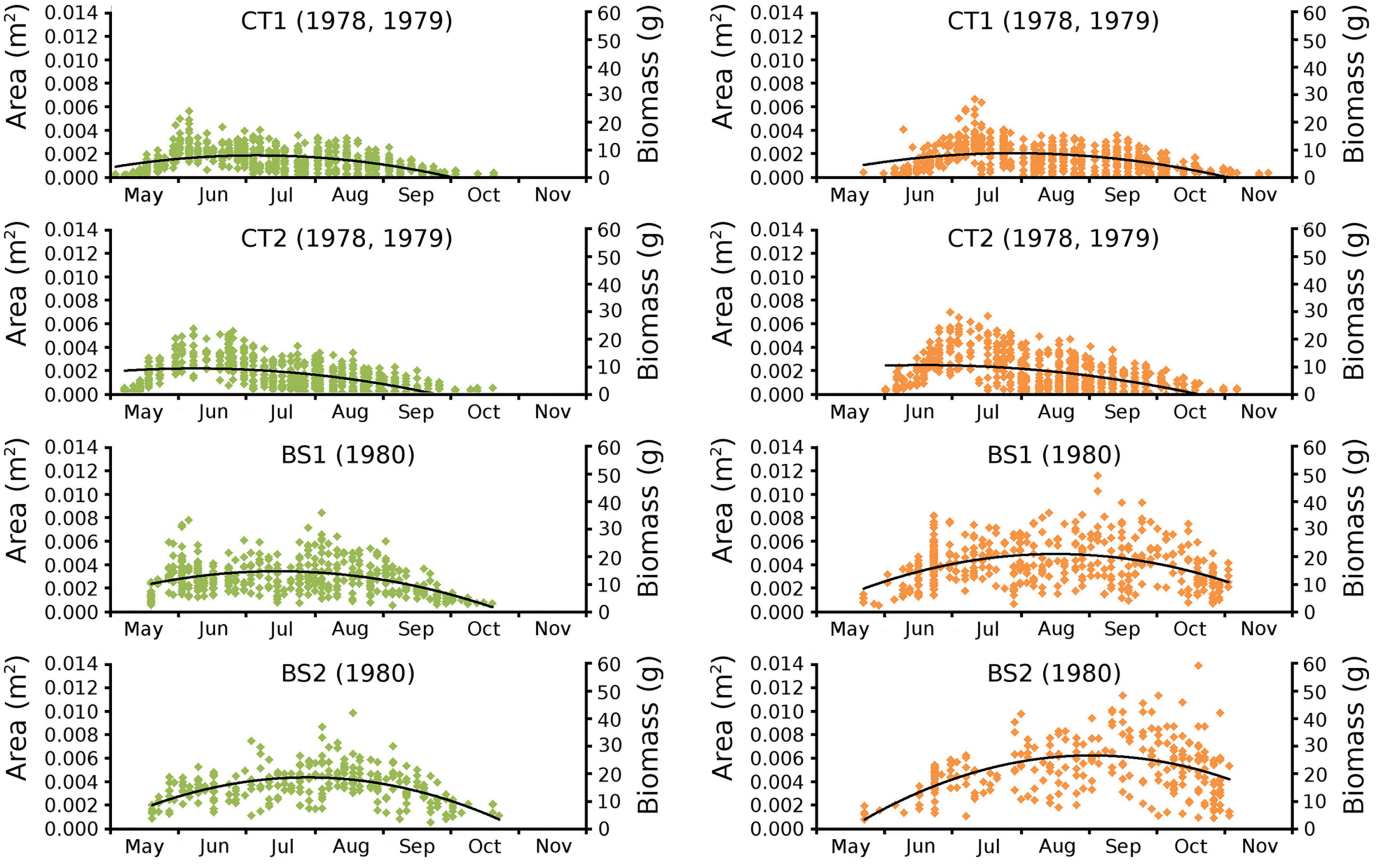
Observed begin and end area of *Nymphoides peltata* leaves. Scatter plots of observed begin (green) and end (brown) area of *Nymphoides peltata* leaves per plot over time with second order curve fitting polynomial. Axes with biomass values are shown on the right side of the plots. See Table 8 for trend line equations with r^2^ values and Table 9 for confidence interval values.

Begin *vs*. end values of leaf area per leaf are shown per plot in scatterplots with trend lines, showing a linear relationship (Fig. 9). Begin and end values of potential leaf area and biomass are shown for all plots over time, indicating growth at the water surface during the vegetation season. Those values are also shown in scatter plots with a second-order polynomial trend line with trend line equations and confidence intervals (Fig. 10, Tables 7 and 8). The trend lines show optima in May/June (CT2), June/July (CT1, BS1) and July/August (BS2) for begin values and in June (CT2), July/August (CT1), August (BS1) and August/September (BS2) for end values.

**Table 7:** Trend line equations for begin and end area (m^2^) and biomass (g AFDW) of *Nymphoides peltata* leaves. Trend line equations for begin and end area (m^2^) and biomass (g AFDW) of *Nymphoides peltata* leaves in plots CT1 (1978, 1979), CT2 (1978, 1979), BS1 (1980) and BS2 (1980). See Table 8 for confidence intervals of the trend lines and Fig. 11 for scatter plots with trend lines.

	Trend line begin area	r^2^	Trend line end area	r^2^
CT1	y = −0.2174x^2^ + 12470x – 2E+08	0.2205	y = −0.1993x^2^ + 11437x – 2E+08	0.1744
CT2	y = −0.1717x^2^ + 9836.8x – 1E+08	0.2372	y = −0.1484x^2^ + 8506.9x – 1E+08	0.1790
BS1	y = −0.3251x^2^ + 19127x – 3E+08	0.1556	y = −0.3868x^2^ + 22782x – 3E+08	0.1438
BS2	y = −0.4737x^2^ + 27882x – 4E+08	0.2685	y = −0.5126x^2^ + 30210x – 3E+08	0.2163
	Trend line begin biomass/annual	r^2^	Trend line end biomass/annual	r^2^
CT1	y = −0.6562x^2^ + 37634x – 5E+08	0.2205	y = −0.6013x^2^ + 34516x – 5E+08	0.1744
CT2	y = −0.5181x^2^ + 29687x – 4E+08	0.2372	y = −0.4479x^2^ + 25674x – 4E+08	0.1790
BS1	y = −1.2760x^2^ + 75075x – 1E+09	0.1556	y = −1.5182x^2^ + 89419x – 1E+09	0.1438
BS2	y = −1.8593x^2^ + 103439x – 2E+09	0.2685	y = −2.0121x^2^ + 118576x – 2E+09	0.2163

**Table 8:** Confidence intervals for begin and end area (cm^2^) and biomass (mg AFDW) of *Nymphoides peltata* leaves. Confidence intervals for begin and end area (cm^2^) and biomass (mg AFDW) of *Nymphoides peltata* leaves in plots CT1 (1978, 1979), CT2 (1978, 1979), BS1 (1980) and BS2 (1980). The biomass is based on annual regression equations. See Table 7 for trend line equations and Fig. 11 for scatter plots and trend lines.

	Leaf area	Mean	St deviation	95% conf int	Lower limit	Upper limit
**CT1**	begin end	1,257.00 1,493.00	840.50 970.22	64.52 74.47	1,192.60 1,418.15	1,321.64 1,567.10
**CT2**	begin end	1,472.00 1,702.00	1,115.58 1,299.33	87.04 101.38	1,885.06 1,600.29	1,559.15 1,803.05
**BS1**	begin end	2,844.66 4,036.37	1,360.42 1,865.72	128.88 176.76	2,715.78 3,859.62	2,973.55 4,213.13
**BS2**	begin end	3,453.00 5,007.00	1,447.83 2,284.98	170.50 269.09	3,282.35 4,737.88	3,623.35 5,276.05
	**Biomass**	**Mean**	**St deviation**	**95% conf int**	**Lower limit**	**Upper limit**
**CT1**	begin end	3,800.00 4,511.00	2,536.64 2,928.12	194.71 224.76	3,605.79 4,286.50	3,995.20 4,736.01
**CT2**	begin end	4,449.00 5,142.00	3,366.82 3,921.37	262.70 305.97	4,186.63 4,836.17	4,712.02 5,448.10
**BS1**	begin end	11,138.72 15,816.19	5,339.64 7,322.96	505.87 693.77	10,632.85 15,122.43	11,644.59 16,509.96
**BS2**	begin end	13,526.00 19,626.00	5,682.72 8,968.55	669.21 1,056.16	12,856.66 18,569.62	14,195.09 20,681.94

Average potential begin and end values of leaf area and leaf biomass for all plots are shown in Table 9.

**Table 9:** Average potential begin and end values for leaf area (cm^2^) and leaf biomass (mg AFDW). Average potential begin and end values for leaf area (cm^2^) and leaf biomass (mg AFDW) of *Nymphoides peltata* in plots CT1 (1978), CT2 (1978), BS1 (1980) and BS2 (1980). See Fig. 10 for scatter plots of begin *vs*. end leaf area.

Plot	Average potential leaf area				Average potential leaf biomass			
	Begin	End	End-Begin	Begin/End	Begin	End	End-Begin	Begin/End
CT1	1,364.12	1,603.05	238.94	0.85	4,123.41	4,844.58	721.17	0.85
CT2	1,511.62	1,711.67	200.04	0.88	4,568.58	5,172.31	603.73	0.88
BS1	2,844.66	4,036.37	1,191.71	0.70	11,138.70	15,816.20	4,677.47	0.70
BS2	3,452.90	5,007.00	1,554.10	0.69	13,526.00	19,626.00	6,099.90	0.69

**Figure 11:**
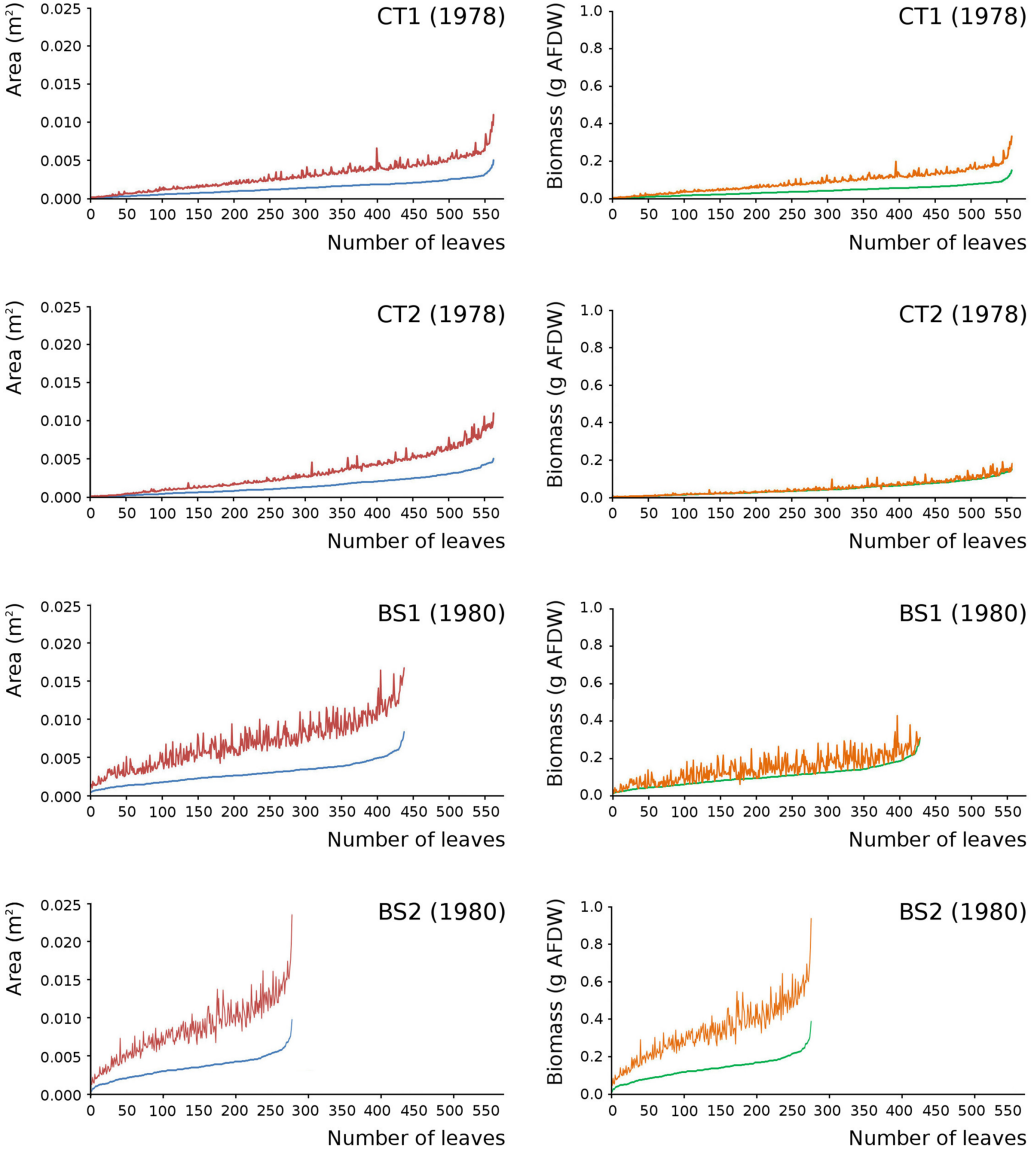
Begin and end values of potential area and potential biomass of all leaves. Begin and end values of potential area and potential biomass of all leaves of *Nymphoides peltata* per plot. All leaves were ranked from low to high begin values. With begin area (blue), end area (red), begin biomass (green) and end biomass (brown). The biomass is computed with the annual regression equation. The leaves are arranged according to their begin values from low to high with related end values.

Leaves in the tank plots were smaller than those in the lake plots (Table 2), and showed a lower increase in total potential leaf area by growth on the surface: 14% for CT1, 12% for CT2, 30% for BS1 and 31% for BS2. The graphs displaying the potential begin and end leaf area (Fig. 11) clearly show differences in growth: the mean growth increase is 18.77% for CT1, 15.62% for CT2, 41.89% for BS1, 45.00% for BS2, respectively. The leaf area also shows that the tank plots contained smaller leaves than the lake plots (Fig. 8).

### Leaf area and biomass

Leaf area data are shown in Table 2. The mean potential leaf area values with standard deviations have been computed over all measuring dates per plot for all leaves, for short shoot leaves and for flowering stem leaves (Table 5).

Leaf biomass data per plot were calculated by monthly and by annual regression equations based on the leaf area of each leaf as calculated from the leaf lengths measured in the plots (Tables 1 and 10).

**Table 10:** Leaf biomass data of *Nymphoides peltata* per plot. Leaf biomass data of *Nymphoides peltata* per plot, computed by monthly and annual regression equations in concrete tanks (CT1, CT2) and Bemmelse Strang (BS1, BS2).

Location Year		CT1 1978	CT2 1978	BS1 1980	BS2 1980
Leaf biomass (monthly)					
Total pot.	g AFDW.m^−2^.yr^−1^	133.24	137.31	272.18	208.59
Max. pot. on date	g AFDW.m^−2^	60.92	58.14	74.32	90.20
Max act. on date	g AFDW.m^−2^	60.89	58.05	63.08	83.91
Max. phot. on date	g AFDW.m^−2^	38.15	38.98	54.70	72.31
Mean pot. per day	g AFDW.m^−2^.d^−1^	0.6596	0.7463	1.6105	1.2342
Mean pot. per leaf	g AFDW.m^−2^	0.0600	0.0615	0.1590	0.1883
Standard deviation	g AFDW.m^−2^	0.0408	0.0513	0.0776	0.0905
Max. pot. date		Jul. 4	Jul. 4	Aug. 26	Aug. 29
Max act. date		Jul. 4	Jul. 4	Aug. 29	Aug. 29
Max. phot. date		Jul. 4	Jul. 9	Aug. 29	Aug. 29
Leaf biomass (annual)					
Total pot.	g AFDW.m^−2^.yr^−1^	106.58	115.45	270.77	213.49
Max. pot. on date	g AFDW.m^−2^	43.18	43.11	69.52	89.07
Max act. on date	g AFDW.m^−2^	43.18	42.79	61.77	80.33
Max. phot. on date	g AFDW.m^−2^	28.78	28.16	55.57	65.68
Mean pot. per day	g AFDW.m^−2^.d^−1^	0.5276	0.6274	1.6022	1.2632
Mean pot. per leaf	g AFDW.m^−2^	0.0480	0.0517	0.1582	0.1927
Standard deviation	g AFDW.m^−2^	0.0299	0.0401	0.0733	0.0898
Max. pot. date		Jul. 11	Jun. 30	Sep. 5	Oct. 20
Max act. date		Jul. 11	Jun. 30	Sep. 5	Sep. 16
Max. phot. date		Jun. 9	Jun. 30	Jun. 23	Sep. 16

**Figure 12:**
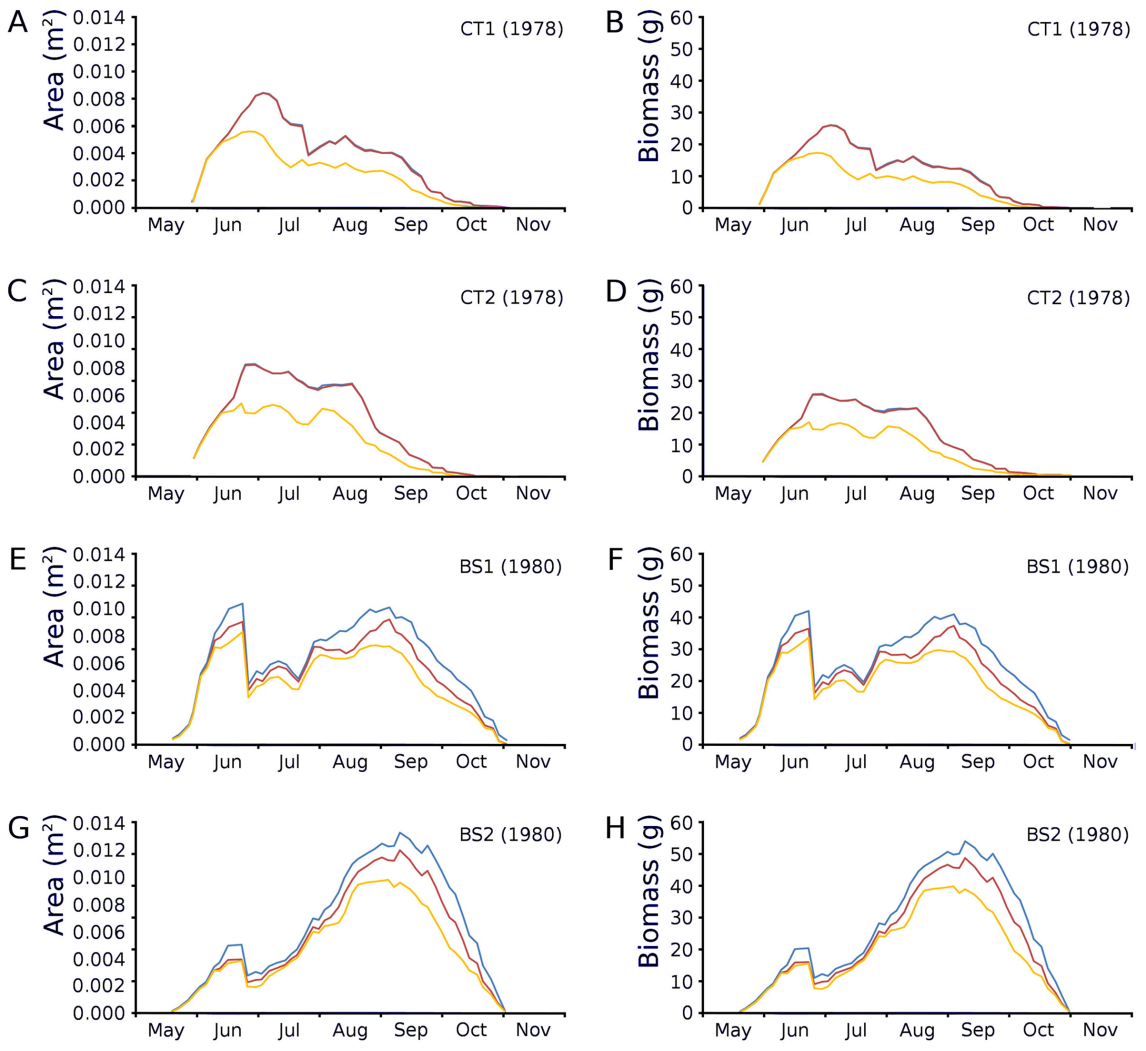
Potential, actual and photosynthetic leaf area (m^2^.m^−2^) and leaf biomass (g AFDW.m^−2^). Potential (blue), actual (red) and photosynthetic (yellow) leaf area (m^2^.m^−2^) and leaf biomass (g AFDW.m^−2^) of *Nymphoides peltata* over time. The left column (A, C, E, G) shows leaf area, the right column (B, D, F, H) shows biomass using annual equations.

Changes over time of potential, actual and photosynthetic leaf area and leaf biomass based on both regression equations demonstrated in the plots CT1 and CT2 a clear peak at the end of June which is maximal for CT1 and CT2 with a lower peak in August after which there is a further decrease. In BS1 and BS2 a clear peak of June is visible, after which a strong loss of leaves is visible due to a flooding after which a second peak was reached in September which was about equal in size with the first peak in the case of BS1 and much higher than that in the case of BS2 (Fig. 12).

### Ratios

Based on leaf characteristics (Table 2), various ratios were calculated for the plots (Table 11) to provide information on the plasticity of the species.

**Table 11:** Ratios based on leaf blade characteristics. Ratios based on leaf blade characteristics of *Nymphoides peltata* in Table 2.

Location Year		CT1 (1978)	CT2 (1978)	BS1 (1980)	BS2 (1980)
Turnover rate, or Vegetation period/Mean leaf life span		7.98	8.17	7.40	5.71
Turnover rate (P/B_max_), or Tot. pot. leaf biomass/Max. pot. leaf biomass		2.47	2.68	3.91	2.40
Total number of leaves/Max. number of leaves		4.20	3.00	4.37	2.92
Mean leaf life span/Total number of leaves	d	0.010	0.009	0.013	0.027
Growth period/Vegetation period		0.847	0.908	0.917	0.935
Leaf area					
Max. pot./Tot. pot.	%	0.4053	0.3736	0.2567	0.4095
Max. act./Tot. pot.	%	0.4051	0.3709	0.2281	0.3693
Max. phot./Tot. pot.	%	0.2701	0.2441	0.2052	0.3020
Leaf biomass					
Max. pot./Tot. pot.	%	0.4051	0.3734	0.2567	0.4172
Max. act./Tot. pot.	%	0.4051	0.3706	0.2281	0.3763
Max. phot./Tot. pot.	%	0.2700	0.2439	0.2052	0.3076
Leaf area/Leaf biomass					
for Max pot.	m^2^/g AFDW	0.0331	0.0331	0.0255	0.0255
For Max. act.	m^2^/g AFDW	0.0331	0.0331	0.0255	0.0255
for Max phot	m^2^/g AFDW	0.0331	0.0331	0.0255	0.0255

The turnover rate Vegetation period/Mean leaf life span was high for CT1, CT2 and BS2, with a very low value for BS2, which may indicate that BS2 suffered least of nutrient limitation and of inundation. The turnover rate Total potential biomass/Maximum potential biomass (P/B_max_) showed similar values for CT1, CT2 and BS2 and a clearly higher value for BS1. Total number of leaves/Maximum number of leaves showed differences between CT1, BS1 and CT2, BS2, which may have been caused by limitation of space and nutrients. Mean leaf life span/Total number of leaves was similar for CT1 and CT2, showed a much higher value for BS1 and a value twice as high for BS2. Growth period/Vegetation period showed similar values for the four plots. Ratios for Leaf area and biomass, *i.e*., Maximum leaf area or biomass (pot., act. and phot.)/Total leaf area or biomass (pot.) generally showed higher values for CT1 and BS2 compared to CT2 and the lowest for BS1. Leaf area/Leaf biomass showed low values for BS1, similar values for CT1 and CT2 and slightly lower values for BS1 and BS2.

## Discussion

### Water temperature and leaf development

The difference in start date of floating leaf development between tank plots and lake plots can be explained by the measurement of water temperature at a depth of 10 cm, because water exchange and the great water mass in the lake delay the warming up of the water at the bottom and therefore the development of leaves from the bottom. In contrast to the growth period of other nymphaeids (*Nuphar lutea* (L.) Sm., *Nymphaea alba* L., *Nymphaea candida* Presl), which ended about halfway the vegetation period (Klok & Van der Velde, 2017), the production of new leaves of *N. peltata* continued over nearly the whole growth period. Growth and vegetation periods showed a high similarity in all plots and seemed to be regulated by water temperature. Development of leaves takes place above a maximum water temperature of 15 °C and at the end of the season leaves disappear when the maximum water temperature drops below 10 °C. Markovskaya et al. (2019) noted the high invasive capacity at high air temperatures at the northern limit of its occurrence with an increase in leaf area.

### Influence of inundations or floods

Inundations, causing a sudden and considerable rise of the water level in the growing season, can diminish the vitality of *N. peltata* or even cause its complete disappearance (Brock, Van der Velde & Van de Steeg, 1987; Yu & Yu, 2011). A rapid increase in water depth has a stronger negative impact than a gradual increase during the growing season (Nohara, 1991; Yu & Yu, 2011). Species with a rapid, adaptive morphological response to fluctuating water levels have continuous and rapid leaf recruitment (Ridge & Amarasinghe, 1984; Yu & Yu, 2011). Leaf life spans of 20–30 days (Tsuchiya, 1991; Kunii & Aramaki, 1992) and a high productivity relative to biomass (Brock et al., 1983a; Twilley et al., 1985; Kunii & Aramaki, 1992) represent a strategy of morphological adjustments to water level fluctuations. *N. peltata* mainly relies on morphological plasticity by petiole elongation to adapt to water level rise, since it cannot escape like free floating plants and it does not develop underwater leaves for survival (Cao & Mei, 2015). Continuous leaf recruitment ensures that young radical leaves, growing and elongating faster than old ones, are always present in the canopy (Ridge, 1985). Nohara (1991) found that biomass of *N. peltata* in sites of more than 1.5 m depth increased in late June and decreased after a flood causing a water level rise of 1 m and lasting 1 week in Lake Kasumigaura. Tsuchiya, Nohara & Iwakuma (1990) found that when leaves were lost by wave action, the plants soon recovered by vegetative growth producing leaves from the runners which are perennial. *Nymphoides peltata* in the Oude Waal showed a low degree of flooding tolerance when a summer flood rose the water level by 3 m with a duration of several weeks. When the rates of elongation and leaf recruitment do not match the rise in water level, plants die prematurely (Brock et al., 1983a; Brock, Van der Velde & Van de Steeg, 1987; Tsuchiya, 1991; Cooling, Ganf & Walter, 2001).

Normally winter and spring floods occur each year outside the vegetation period in Bemmelse Strang after which the water level gradually decreases without influence on later development of *N. peltata* (Brock, Van der Velde & Van de Steeg, 1987). Paillisson & Marion (2011) noted that early spring water level fluctuations have a poorly related influence on the biomass of *N. peltata* in contrast to *Nymphaea alba* L. and *Nuphar lutea* (L.) Sm. The leaf loss by the early summer inundation of lake plots BS1 and BS2 during this research was compensated by *N. peltata* by developing new leaves of which the petioles adapted to the new water levels. In the case of border plot BS2 the inundation also led to an increased growth nearly till the end of the vegetation period (Growth period/Vegetation period 93.5%).

### Impact of nutrient availability

This research further investigated the consequences for leaf blade characteristics and leaf development from the posed hypothesis that dense enclosed *Nymphoides* stands recycle nutrient sources more frequently than less dense open stands. High consumption of nutrients by growth may lead to limited resources for further development and growth, which influences leaf development. Experiments with herbaceous terrestrial plants under conditions of lowered nutrient levels demonstrated that plants developed smaller leaves with a shorter leaf life span, leading to a higher leaf turnover, a lower biomass and the development of more flowers (El Hinnawy, 1956). Brock et al. (1983b) studied the nitrogen and phosphorus accumulation and cycling in and outside a *N. peltata* stand in Bemmelse Strang during 1980 and found that during the growing season most of the nutrients were translocated to petioles and leaves, that uptake of nutrients was through the roots from the interstitial water of the sediment and that phosphorus was taken up for at least 80% from the sediment. Harvesting of *N. peltata* has a positive effect by decreasing the total nitrogen, ammonium nitrogen and chemical oxygen demand, though it may also result in an increase of phosphorus causing algal blooms (Zhu et al., 2019).

### Plot ranking by nutrient availability

Leaf development of *N. peltata* was followed in four plots representing different situations that may influence nutrient availability: volume of growing area, thickness of rooting layer, water depth, water exchange and competition with other macrophyte species. The tanks with plots CT1 and CT2 provided a limited volume for growing, while the lake with plots BS1 and BS2 had an “unlimited” volume. The average depth of CT1, CT2 and BS1 was similar (45–67 cm), in contrast to BS2 (117 cm). BS1 had a thicker detritus layer compared to BS2, which may have enlarged the nutrient availability for BS1. Water flow in the lake as well as the unexpected inundation by river water will have improved the nutrient availability in the water layer for BS1 and BS2. The center plot BS1 of the lake stand had a much thicker vegetation than border plot BS2. In contrast with the monospecific *N. peltata* vegetation in CT2, CT1 contained a mixed culture of *N. peltata* and some helophytes (*Glyceria* spp.). Less nutrient availability is expected for *N. peltata* in CT1 due to competition with the helophytes. Considering these differences, the ranking of the plots from low to high nutrient availability was expected to be CT1, CT2, BS1, BS2.

### Plot ranking for characteristics

An increasing order was found for maximum leaf life span and maximum leaf length, and a decreasing order for number of leaves, number of short shoot leaves, new leaves per day, and the duration of the vegetation period. Comparing CT with BS plots showed higher CT-values for flowering stems, maximum number of leaves, duration growth period, duration flowering stem period, and lower CT-values for maximum leaf length, minimum leaf length, begin and end leaf length, leaf area, leaf biomass.

The total number of leaves per plot showed a decreasing order for the expected ranking. However, if a distinction between short shoot leaves and flowering stem leaves is made, the number of short shoot leaves showed a similar order: CT1, CT2 and BS1 (about the same), BS2, but the number of flowering stem leaves showed a large difference between tank and lake plots and a different order: CT2 (848), CT1 (404), BS1 (48) and BS2 (12). The difference in flowering stem leaves between CT1 and CT2 might be caused by the mixed culture in CT1 leaving less volume for development of long shoots. This means more leaves and more flowers are an indication of nutrient shortage.

The total potential leaf area of plots, a measure for leaf production, shows the increasing order CT1, CT2, BS2, BS1. The total potential biomass shows the same sequence using both monthly and annual regression equations for tank and lake plots. Since the annual equations are “averaging”, the monthly equations clearly show a higher biomass production in July for CT1 and CT2, while, as a result of the flooding of June 23 in BS1 and BS2, only a slightly higher production is shown in these plots. Consequence of nutrient limitation may be the production of smaller leaves and less biomass.

The maximum leaf life span increased with the expected ranking (CT1, CT2, BS1, BS2), but the mean leaf life span shows the order CT2 and BS1 (about the same), CT1, BS2 from low to high. Here, sexual reproduction might be an important factor that reduces the leaf life span, since CT2 and BS1 showed more flowering stems than CT1 and BS2. Although the trend is clear, the life span frequency of all leaves showed no significant differences between the plots (single-factor ANOVA test; *p* ≥ 0.05), so leaf life span frequency can be considered a stable trait.

### Differences between plots

Wu & Yu (2004) and Wu et al. (2006) found in microcosm experiments that at increasing competition by rice (*Zizania latifolia* (Griseb.) Turcz ex Stapf), *N. peltata* showed lower leaf numbers, leaf areas and water surface coverage. Such effects on *N. peltata* due to the presence of *Glyceria* in CT1 were not observed in the tank plots, as differences in leaf blade characteristics of the two CT plots were not significant (Table 2). A possible explanation might be that competition intensity was not strong enough to make *N. peltata* produce morphological differences, apart from the lower number of flowering stem leaves compared to CT2.

The peak density of leaves in the tank plots was reached in August and in Bemmelse Strang in June (center plot, BS1) and September (border plot, BS2), respectively. After the peak in June and the flooding, the production of new short shoot leaves in BS1 had to compete with the development of flowering stems caused by nutrient shortage by enclosed growth, which may have prevented a higher peak later in the season. The flooding had less impact on the number of leaves in BS2, which reached a later and higher peak in September, also while there were no limiting nutrients at the border of the stand as the bare sediment could be colonized without competition.

Consequence of nutrient limitation may be the development of more, but smaller leaves.

Mean leaf length was similar in the tank plots, but much larger in the lake plots, where less, but larger leaves and also less flowering stem leaves developed. The largest leaves occurred in border plot BS2. The leaf area in the tank plots showed a rapid increase to a peak level after which a lower level was established, an indication of nutrient shortage preventing a further increase.

Leaf area and biomass in the lake plots reached a later peak (September), compared to the tank plots (July). The late peaks in leaf area and biomass of border plot BS2 were relatively very high. This might be explained by the lower impact of the June 23 inundation at BS2, compared to BS1 as in the latter plot the peak of leaf area and biomass in June was already very high when the flooding started. In BS1 the later peak in leaf area and biomass in September was even lower than in June. The graphs displaying the potential biomass for begin and end leaf size show very small differences between begin and end biomass values for CT2 and BS1. This might be due to lack of nutrients resulting in mainly growth by cell stretching causing low leaf biomass values. The decrease after the early optima for CT1 and CT2 may indicate the growing influence of nutrient limitation in time.

The high leaf area and biomass production in border plot BS2 can be explained by a higher nutrient availability and room for expansion at the border of the stand favoring vegetative growth, while in center plot BS1 high densities of short shoots and roots lead to competition of plant organs for nutrients inducing sexual reproduction by producing flowering stems with flowers at the expense of leaf production. The high production of flowering stems is shown clearly in the tank plots and can be the result of nutrient poor conditions as found for terrestrial plants (El Hinnawy, 1956).

Comparing CT with BS plots showed higher CT-values for flowering stems, maximum number of leaves, duration of growth period, duration flowering stem period, and lower CT-values for maximum leaf length, minimum leaf length, begin and end leaf length, leaf area, leaf biomass.

### Ratios

Based on leaf blade characteristics, various ratios were calculated for the plots. The turnover rate Vegetation period/Mean leaf life span was high for CT1, CT2 and BS2, with a very low value for BS2, which may indicate that BS2 suffered least of nutrient limitation and of inundation. The turnover rate Total potential biomass/Maximum potential biomass (P/B_max_) showed similar values for CT1, CT2 and BS2 and a clearly higher value for BS1. Total number of leaves/Maximum number of leaves showed differences between CT1, BS1 and CT2, BS2, which may have been caused by limitation of space and nutrients. Mean leaf life span/Total number of leaves was similar for CT1 and CT2, showed a much higher value for BS1 and a value twice as high for BS2. Growth period/Vegetation period showed similar values for the four plots. Ratios for Leaf area and biomass, *i.e*., Maximum leaf area or biomass (pot., act. and phot.)/Total leaf area or biomass (pot.) generally showed higher values for CT1 and BS2 compared to CT2 and the lowest for BS1. Leaf area/Leaf biomass showed low values for BS1, similar values for CT1 and CT2 and slightly lower values for BS1 and BS2. From the differences in ratios it can be concluded that the ratio growth/vegetation period is most stable over the plots, which indicates that this is a characteristic of the plant species. Unfortunately, we have data of only four plots, which is insufficient to allow further generalizations based on the ratios. We need wide scale investigations to interpret these results.

## Conclusions

The results of this study lead to the following conclusions:

(a) Growth and vegetation periods of *N. peltata* seemed to be regulated by water temperature (10–30 °C): growth stops when the water temperature drops below 10 °C and this indicates a stable trait.
(b) The growth period started in May and ended mid-October with continuous production of floating leaves during nearly the whole vegetation period indicating its capacities for rapid colonization and spread in contrast to waterlilies. The petioles of the floating leaves adapt to the water level. The water level in the tanks was very stable, but the inundation of the lake plots caused the sudden loss of existing leaves and a lower production of new leaves for about a month, then followed by an increase. The deeper border plot suffered less from the inundation and showed an absolute leaf maximum in September.
(c) Resources in the tanks were limited compared to the lake which resulted in the production of a very high number of smaller leaves with lower totals for leaf area and biomass and in abundant sexual reproduction *via* flowering stems.
(d) Turnover rates and other ratios calculated, appeared to be relatively similar for plots CT1, CT2 and BS1, but for the deeper border plot BS2 lower ratios were found.
e) All these results suggest that established dense enclosed stands, have to recycle nutrients more frequently than stands in pioneer or border situations with consequences for leaf development and leaf blade characteristics.

## Supplemental Information

10.7717/peerj.13976/supp-1 Supplemental Information 1 Data 1978 Concrete Tank 1.

10.7717/peerj.13976/supp-2 Supplemental Information 2 Dataset 1978 Concrete Tank 2.

10.7717/peerj.13976/supp-3 Supplemental Information 3 Dataset 1980 Bemmelse Strang plot 1.

10.7717/peerj.13976/supp-4 Supplemental Information 4 Dataset 1980 Bemmelse Strang 2.

## Additional Information and Declarations

### Data Availability

The following information was supplied regarding data availability: The raw data is available in the Supplemental File.
